# Guidelines for diagnosis and management of congenital central hypoventilation syndrome

**DOI:** 10.1186/s13023-020-01460-2

**Published:** 2020-09-21

**Authors:** Ha Trang, Martin Samuels, Isabella Ceccherini, Matthias Frerick, Maria Angeles Garcia-Teresa, Jochen Peters, Johannes Schoeber, Marek Migdal, Agneta Markstrom, Giancarlo Ottonello, Raffaele Piumelli, Maria Helena Estevao, Irena Senecic-Cala, Barbara Gnidovec-Strazisar, Andreas Pfleger, Raquel Porto-Abal, Miriam Katz-Salamon

**Affiliations:** 1 Hôpital Universitaire Robert Debré, Centre de référence des maladies respiratoires rares, and Université de Paris, Paris, France; 2 Staffordshire Children’s Hospital, Stoke-on-Trent, Staffs and Great Ormond Street Hospital, London, UK; 3 Istituto Giannina Gaslini, UOSD Laboratory of Genetics and Genomics of Rare Diseases, Genoa, Italy; 4 Department of Pediatrics, Klinikum Dritter Orden, Munich, Germany; 5 Niño Jesús University Children’s Hospital, Pediatric Intensive Care Unit, Madrid, Spain; 6 Department of Anaesthesiology and Intensive care, Children’s Memorial Health Institute, Warsaw, Poland; 7 Karolinska University Hospital, Stockholm, Sweden; 8 Pediatric Primary Care, ASL3, Genoa, Liguria Italy; 9 Sleep Disordered Breathing and SIDS Center, Meyer Children’s Hospital, Florence, Italy; 10 Pneumology Department, Pediatric Hospital of Coimbra, Coimbra, Portugal; 11 University Hospital Centre, Department of Pediatrics, Zagreb and School of Medicine, Zagreb, Croatia; 12 University Children’s Hospital, Department of child, adolescent & developmental neurology, University Clinical Centre Ljubljana, Ljubljana, Slovenia; 13 Medical University of Graz, Paediatric Pulmonology and Allergology, Graz, Austria; 14 Department of Pediatrics, Puerta de Hierro Hospital, Madrid, Spain

**Keywords:** Central hypoventilation, Dysautonomia, Hirschsprung disease, Neural crest tumour, Long-term ventilation, PHOX2B

## Abstract

**Background:**

Congenital Central Hypoventilation Syndrome (CCHS) is a rare condition characterized by an alveolar hypoventilation due to a deficient autonomic central control of ventilation and a global autonomic dysfunction. Paired-like homeobox 2B (PHOX2B) mutations are found in most of the patients with CCHS. In recent years, the condition has evolved from a life-threatening neonatal onset disorder to include broader and milder clinical presentations, affecting children, adults and families. Genes other than PHOX2B have been found responsible for CCHS in rare cases and there are as yet other unknown genes that may account for the disease. At present, management relies on lifelong ventilatory support and close follow up of dysautonomic progression.

**Body:**

This paper provides a state-of-the-art comprehensive description of CCHS and of the components of diagnostic evaluation and multi-disciplinary management, as well as considerations for future research.

**Conclusion:**

Awareness and knowledge of the diagnosis and management of this rare disease should be brought to a large health community including adult physicians and health carers.

## Introduction

“Primary alveolar hypoventilation”, also referred to as “Ondine’s curse”, was first reported in a newborn in 1970 [59]. It was defined as “alveolar hypoventilation due to an abnormality in the automatic control of ventilation by the central nervous system”, not explained by any pulmonary, cardiovascular, neurologic or muscular anomalies [59]. The name of Congenital Central Hypoventilation Syndrome (CCHS) was given to the disease in 1978 by authors who used phrenic nerve stimulation as a treatment option [41]. CCHS (2020-ICD-10-CM and ICSD3 code: G47.35; MIM 209880, ORPHA 661) is a rare condition, the incidence of which has been estimated to be at 1/148,000–1/200,000 live births [84, 92], and the prevalence at 1/500,000 individuals [92]. Clinical hallmarks of CCHS are well-described: central hypoventilation due to abnormally reduced or absent ventilatory responses to hypercapnia and hypoxia and associated manifestations of autonomic dysfunction such as Hirschsprung disease (HD) and neural crest tumours [32, 106].

*PHOX2B* was identified as the major CCHS-causing gene for patients in 2003 [2]. CCHS is currently classified as a “sleep-related alveolar hypoventilation” and “presence of mutations of *PHOX2B* gene” by the International Classification of Sleep Disorders version 3 published in 2014 [80].

However, the nature of the disease has evolved over the last two decades, moving from a neonatal high mortality-risk disease towards less severe and much broader clinical presentations. Most cases manifest in the neonatal period, but increased later-onset presentations are recognized during childhood and adulthood. Familial cases with CCHS exist [22, 45, 46, 48, 53, 86, 95], due to parental transmission of somatic and germline mosaic mutations [6, 9, 72]. In the meantime, *PHOX2B*-mediated mechanisms have been extensively investigated and better understood in vitro [4, 5, 7, 8, 20, 24, 25, 68, 97], in vivo [27, 44, 70], and in CCHS patients [28, 33, 92–94, 99]. However, most pathogenic pathways are yet to be disclosed. In addition, a subset of patients with a CCHS phenotype has no mutation in *PHOX2B*. Recently, mutations of two new genes, *MYO1H* and *LBX1*, have been found in two consanguineous CCHS families [39, 88]. It is expected that other genes may account for the patients without any apparent causative *PHOX2B* mutation. Finally, although some molecules have shown effects on ventilation e.g. carbamazepine [81], desogestrel [89], CCHS currently has no curative treatment. Management is still supported by lifetime assisted ventilation and multi-disciplinary care. Research studies focus to target sensitive molecules for a putative pharmacologic treatment.

## Methods and objectives

The Guideline Development Group (GDG) was a product from the European CCHS Consortium Project, funded by the EU Executive Agency for Health and Consumers (EAHC grant 2008 12 06). The GDG first requested a literature search, which was carried out by the Centre for Reviews and Dissemination at the University of York, UK. Members of the GDG then carried out further searches, concentrating on their own topics.

Details of the search strategy are given in Additional file 1. As CCHS is a rare disease, most of the evidence is descriptive and of low grade of recommendation. This draft was based on the published evidence where possible and then combined with clinical expertise as required. The resulting draft and recommendations therefore was a blend of published evidence and clinical experience. The manuscript has been sent to a group of international specialists (see the acknowledgements section) then further amended before a final review by all members of the GDG.

These guidelines aim to present the evidence base for the diagnosis, follow-up and treatment of infants, children and adults with CCHS. As discussed above, the clinical features of the condition continue to change and these guidelines must be read within that context. For the purpose of the present guidelines, CCHS will be defined using its original definition, i.e. “an alveolar hypoventilation due to an abnormality in the automatic control of ventilation by the central nervous system”, not explained by any pulmonary, cardiovascular, neurologic or muscular anomalies. The guidelines also highlight areas where research is needed.

## Diagnosis

### Diagnostic criteria for CCHS

**Fig. 1:**
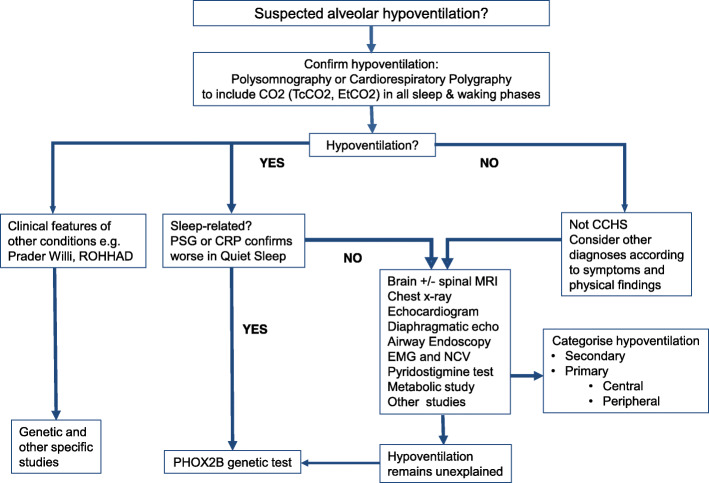
Diagnostic algorithm for alveolar hypoventilation. Abbreviations: CCHS, Congenital Central Hypoventilation Syndrome; CRP, cardiorespiratory polygraphy; EMG, electromyogram; Et-CO_2_, end-tidal CO_2_; MRI, Magnetic Resonance Imaging; PHOX2B, Paired-like homeobox 2B; PSG, polysomnography; ROHHAD, Rapid-onset obesity with hypoventilation, hypothalamic dysfunction, and autonomic dysregulation; NCV, nerve conduction velocity; Tc-CO_2_, transcutaneous PCO_2_

CCHS is defined as an alveolar hypoventilation due to due to a deficient autonomic central control of breathing and a global autonomic dysfunction. The Fig. 1 shows the diagnostic algorithm for diagnosis of alveolar hypoventilation.

In CCHS, polysomnography shows hypoventilation (hypercapnia and hypoxemia) which is typically more severe during sleep than during wakefulness, and most severe during Quiet Sleep (or Non Rapid Eye Movement sleep, NREM) than during Active Sleep (or Rapid Eye Movement sleep, REM). The classical pattern is a reduction in the respiratory rate and in the amplitude of airflow and tidal volume. Central apnoeas can be observed. Abnormally low or absent ventilatory responses to hypercapnia and to hypoxia are present at both sleep and wake states [59].

At diagnosis, a thorough assessment of spontaneous ventilation during REM and NREM sleep, as well as during wakefulness should be performed under medical supervision, aiming to estimate the severity of hypoventilation during wakefulness and each sleep stage. These data are essential for respiratory management for each individual patient.

### CCHS should be characterized by

- Age at presentation: (i) neonatal onset (in the first month of life), (ii) later onset (presentation after 1 month of age, or in childhood or adulthood)
- Severity of the respiratory phenotype: (i) sleep hypoventilation; (ii) 24 h per day hypoventilation
- Presence of other conditions: (i) CCHS with Hirschsprung disease (known as Haddad Syndrome), (ii) CCHS with neural crest tumours, (iii) CCHS with Hirschsprung disease and neural crest tumours.
- Type of gene anomalies: (i) with PHOX2B anomalies: PARMs, NPARMs, or PHOX2B deletion, (ii) with other gene mutations, (iii) no identified mutations.

### Main presenting symptoms of CCHS

are commonly features of chronic or acute hypoventilation. In most cases, apnoeas and hypoventilation may occur at birth, or less frequently during childhood or adulthood. Less common are presentations including brief resolved unexplained events (BRUE), repeated oxygen desaturation, severe central sleep apnoeas, failure to wean from a ventilator after pneumonia, sleep hypoventilation more severe than expected for obstructive sleep apnoeas, acquired pulmonary hypertension, delayed recovery from anaesthesia or opioids, coma after sedatives, or near drowning [106]. Familial cases are diagnosed by genetic tests performed after an affected individual has been identified.

### Definition of alveolar hypoventilation

Normal values of blood gases vary with age and methods used. Arterial partial pressure of carbon dioxide (PaCO_2_) between 4.7–6.0 kPa (35–45 mmHg) and arterial oxygen saturation (SaO_2_) ≥ 95% are considered as normal (Table 1).

**Table 1:** Reference values for PO_2_, oxygen saturation and PCO_2_

***Measurement***	***Reference values***	***Comments***
***Oxygenation***		
Arterial oxygenation (SpO_2_)	≥ 95%	In room air / no supplemental O2 Ensure there is good detection of pulse waveforms to exclude movement artefacts or inadequate pulse detection
Transcutaneous PO_2_ (TcPO_2_) Arterial/Capillary PO_2_	80–100 mmHg 10.7–13.3 kPa	In-hospital use only Change site of the heated probe according to manufacturer’s recommendations
***Carbon Dioxide***		
Transcutaneous (TcPCO_2_)/ Arterial/Capillary PCO_2_	35–45 mmHg 4.7–6.0 kPa	Change site of the heated probe according to manufacturer’s recommendations
End-tidal CO_2_ (ETCO_2_)	30–40 mmHg 4.0–5.3 kPa	Measured at the nose or tracheostomy cannula Technically difficult, requires end-tidal plateau

For children, hypoventilation during sleep is defined as a PCO_2_ > 6.7 kPa (50 mmHg) during > 25% of total sleep time (AASM 2012). Methods include prolonged and continuous non-invasive measurement of transcutaneous/end-tidal PCO_2_ (and transcutaneous oxygen saturation), preferably confirmed with blood gases. For adults, hypoventilation during sleep is defined as a PaCO_2_ > 7.3 kPa (55 mmHg) for ≥10 min or an increase in PaCO_2_ during sleep ≥1.3 kPa (10 mmHg) in comparison to awake supine values and a value > 6.7 kPa (50 mmHg) for ≥10 min (AASM 2012). Hypoventilation during wakefulness is usually defined as PaCO_2_ equal to or greater than 6.0 kPa (45 mmHg).

### Main differential diagnoses for CCHS

Hypoventilation can derive from peripheral or central causes. Central hypoventilation may be secondary to use of drugs or to central nervous system diseases such as cerebrovascular accidents, trauma, tumours, bone compression (e.g. Chiari malformation). (Fig. 1).

Central hypoventilation is called “primary” if the disease has its origins in the brainstem respiratory centres. Primary Central Hypoventilation Syndromes present a wide spectrum of clinical manifestations, (i) either in association with a clinically and genetically well-determined disease (e.g. Prader Willi Syndrome, Familial Dysautonomia etc), (ii) or still remaining within the framework of clinical entities yet to be completely characterized (e.g. Obesity Hypoventilation Syndrome, ROHHAD, etc). ROHHAD, a very rare condition, stands for Rapid Onset Obesity, Hypoventilation, Hypothalamic and Autonomic Disorder. Affected subjects typically present with rapid onset of obesity during early childhood; other key features develop later and include hypoventilation, endocrinopathies (water imbalance, precocious puberty), autonomic dysregulation (strabismus and disorders of temperature control) and sudden cardio-respiratory arrest. Specialist review and some basic investigations usually help exclude these other conditions.

### The following patients should undergo genetic study of PHOX2B (see the Fig. 1)

- infants with central hypoventilation at birth or in the first month of life
- patients with unexplained central hypoventilation manifesting after the first month of life
- hypoventilation manifesting in the setting of general anaesthesia or sedative use
- adults presenting with unexplained central sleep-related hypoventilation
- patients affected by Hirschsprung disease and hypoventilation
- patients presenting with neural crest tumours and hypoventilation
- patients presenting hyperinsulinism in association with hypoventilation
- any child born to a parent with known CCHS
- parents of children with CCHS (see the section “risk of recurrence”).
- patients with hypoventilation associated with rapid onset obesity and hormonal disturbance

We recommend that patients with BRUE or central sleep apnoeas undergo at least prolonged and continuous PCO_2_ monitoring to exclude hypoventilation if possible before discussion for PHOX2B testing. We do not advocate testing for PHOX2B in patients with apnoeas or BRUE and without hypoventilation.

### PHOX2B gene anomalies are found in most of the patients with CCHS

Different mutations of the PHOX2B gene (mostly heterozygous) have been reported: in-frame tandem duplications of tracts of different lengths of the polyalanine stretch in the exon 3 (polyalanine repeat mutations, PARM) are the most frequent [2, 58, 79, 102]. Specific techniques designed to amplify GC-rich regions are needed to detect PARMs, and especially the largest expansions which are prone to escape amplification. The number of repeats is normally set at 20 repeats, but in the PHOX2B polyalanine expansion 24 to 33 repeats are present on the affected allele in patients with CCHS [2, 3, 12, 15, 29, 52, 58, 67, 73, 79, 96, 97, 102].

Less common are non-PARMs (NPARMs) including missense, nonsense, and frameshift mutations. More rarely, CCHS-compatible phenotypes, including isolated Hirschsprung disease and BRUE and symptoms suggestive of autonomic dysregulation, can be found in association with either chromosomal rearrangements, mainly large deletions, involving the whole PHOX2B locus [43] or contractions of the polyalanine PHOX2B tract [24], thus suggesting the phenotypic spectrum of PHOX2B defects is quite wide and is not yet completely defined.

### Do the PHOX2B anomalies predict the clinical presentation?

An association between polyalanine expansion length and severity of autonomic dysfunction has been proposed [58, 102]. A similar relationship is described between the genotype for polyalanine mutations and the need for continuous ventilatory dependence, although this is inconsistent [52]. Later-onset cases usually have the 20/24 or 20/25 genotype with milder hypoventilation. However, this relationship is variable and a few remarkable exceptions are known at present [46].

Patients with NPARMs manifest a wide spectrum of phenotypes, some of whom may have only mild hypoventilation, while others have extensive gut involvement, need for continuous ventilatory support, and increased tumour risk over 1 year of age [15]. Hypoventilation may not be the main feature in some PHOX2B mutations [19].

For both PARMs and NPARMs, a wide variability in mutation penetrance and expressivity is emerging. While this phenomenon is still limited for PARM (few cases are reported with phenotypes unexpected according to polyalanine length), differences for NPARM have been recognized both within and between missense, nonsense, and frameshift mutation groups [45]. The pathogenicity level and therefore the severity degree of these latter mutations can be predicted, as recently proposed, according to the kind of shift induced by the indel change, namely whether the reading frame of the mRNA translation is moved forward of one or two nucleotide positions [23].

### The risk of recurrence for CCHS

can only be defined for PHOX2B mutation positive cases. Transmission is autosomal dominant with a variable penetrance. A large proportion of asymptomatic parents of patients with CCHS do not carry any mutation of the PHOX2B gene, suggesting that most of the affected patients present a de novo mutation, arising during the post-zygotic period or in one of the two gametes (germline mosaicism cannot be demonstrated unless a second child is born from an asymptomatic, non-carrier couple).

A proportion of unaffected parents have turned out to carry either a low penetrant PHOX2B mutation in all their cells, being carriers of constitutive or germline mutations, or a fully penetrant PHOX2B mutation in a proportion of their cells, showing somatic mosaicism of the genetic defect. Mosaicism can be demonstrated for somatic cells, but not for germ cells. Individuals with a mosaicism cannot have inherited the mutation from their parents and for this reason; no antecedents are expected to carry the mutation. Parents negative for both constitutive and mosaic PHOX2B mutations may still have a germline cell mutation (germinal mosaicism) and for this reason, may be advised to undergo prenatal genetic testing. Thus, in parents who are carriers: non-mosaic (constitutive) carrying parents have a 50% risk of transmitting the mutation to their children; proven somatic mosaicism is associated with a reduced (less than 50%) but not quantifiable risk of recurrence.

CCHS patients with mutations of PHOX2B gene have a 50% risk for transmitting the mutation to their children (autosomal dominant inheritance). The offspring who receives the mutation is affected to a degree that depends on the penetrance of the mutation transmitted; for example, incomplete penetrance or very mild manifestations have been demonstrated for the 20/24 or 20/25 genotypes and also for most of the missense, nonsense, and a few frameshift mutations.

### Can CCHS occur without PHOX2B anomalies?

Recently, mutations of two new genes, *MYO1H* and *LBX1*, have been found in two consanguineous families with CCHS [39, 88]. However, there are yet unknown genes that affect the development of neurons responsible for breathing control. There is a subset of patients with a CCHS phenotype and without any identified gene mutations.

A number of genetic anomalies were found such as mutations of protein-altering mutations in receptor tyrosine kinase (RET), and endothelin converting enzyme 1 (ECE1) [102].

### A comprehensive assessment of autonomic dysfunction

should be undertaken at diagnosis, involving mainly the cardiovascular, digestive and ocular systems, as well as the metabolic and endocrine status. The neurodevelopmental and neurocognitive status should be assessed. Brain MRI was performed at confirmation of central hypoventilation to rule out other differential diagnoses prior to the availability of a genetic diagnosis. Brain MRI is usually normal but associated brain anomalies have been reported as co-occurring with CCHS [10]. Investigation for neural crest tumours should be discussed, depending on patient’s age and genotype.

## Respiratory management

### Respiratory assessment

Patients with CCHS typically have no overt clinical signs of hypoventilation, do not perceive sensations of dyspnoea, hypoxia and/or hypercapnia, and do not increase respiratory rate or effort in response to hypoxia. Respiratory assessment should be performed at regular intervals during lifetime. It aims to assess ventilation and blood gases status during spontaneous and assisted ventilation, both when awake and asleep. Measurements should include prolonged continuous recordings by a polysomnogram or alternative cardio-respiratory recording including PCO_2_ measurement and ensuring sufficient sleep cycles and awake states are recorded. Check-ups should be performed at least annually, or more frequently in young children due to age-dependent physiological changes (Table 2).

**Table 2:** Follow up programme

Functions	Subjects	Frequency	Test	Objective of testing
**Spontaneous and assisted ventilation**	All patients	< 2yo: every 2–6 months ≥2yo: annually As often as needed if symptoms	-Wakefulness: SpO_2_, Tc or Et-CO_2_ -PSG or CRP with SpO_2_, Tc or Et-CO_2_	-Assess spontaneous breathing while awake -Adjust ventilator settings during sleep
**Tracheostomy**	With tracheostomy	-If symptoms (desaturation, pain, bleeding, breath holding spells, recurrent infections, intolerance to speaking valve or plugged tracheostomy, change in voice) -After changing the tube size or type -Before decannulation -Every 3–6 months in children in the first 2 years after tracheostomy	-Tracheo-bronchoscopy -Simple fibre-optic tracheoscopy	-Detect tracheostomy-related complications -Check the position of the tube tip (simple fibre-optic tracheoscopy instead of bronchoscopy)
**Maxillo-facial growth**	With mask ventilation	-Every 4–6 months -Annually for older patients	-Examination by maxillo-facial specialist -Imaging if needed	-Detect midface deformation
**Cardiovascular system**	All patients	Annually, and as often as needed if symptoms	−48-72 h ECG Holter -24 h BPAM -Echocardiogram	-Detect arrhythmias -Detect complications of ineffective ventilation
**Response to physical exertion**	>6yo	Annually to every 2–3 years (in patients breathing spontaneously while awake)	Exercise test with bike or treadmill	Verify response of SpO_2_/ CO_2_ to physical effort
**Digestive system**	All patients	Each visit	Symptoms, physical examination anthropometry,	Detect Hirschsprung, oesophageal and large bowel dysmotility
**Eyes**	All patients	<6yo: Annually >6yo: According to ophthalmologist	Comprehensive ocular testing	-Detect visual disorders -Adapt glass or lens correction
**Neurological development**	All patients	< 2-3yo: Every 4–6 months >6yo: Every 2 years As often as needed if disorders	Comprehensive neurocognitive tests	-Detect neurocognitive disorders -Assess education needs
**Endocrinology and Metabolism**	All patients	Once, then as needed	- 24 h glycaemia -OGTT	Identify risk of hypo or hyperglycaemia
**Neural crests tumours**	−20/28–20/33 PARMs -NPARMs	<2yo: Every 6 months 2-7yo: Annually or bi-annually >7yo: according to local oncologist protocols	-Chest and abdominal imaging, KUB ultrasound -Total body MRI if needed	Detect neural crests tumours
**Autonomic dysregulation**	>6yo	If symptoms	Testing: tilt testing, deep breathing, Valsalva maneuver, thermal stressors	Assess autonomic dysregulation

Abbreviations: *BPAM* blood pressure ambulatory monitoring; *CRP* cardio-respiratory polysomnography; *ECG* electrocardiogram; *Et-CO*_*2*_ end-tidal CO_2_; *KUB* kidney, ureter and bladder X-ray; NPARM non-PARM; *OGTT* oral glucose tolerance test; *PARM* polyalanine repeat mutation; *PSG* polysomnography; Tc, transcutaneous

### Ventilatory support is life support for patients with CCHS

At present, no medications have been shown to increase ventilation sufficiently and with sustained action to prevent patients with CCHS from assisted ventilation. Thus, ventilatory support should be provided, aiming to prevent the deleterious consequences of even mild hypoventilation and especially on neurocognitive development. For each individual patient, ventilatory support should allow adequate ventilation at any time, during sleep and wakefulness if needed, during growth, with changes in activities and during intercurrent events. A patient who needs night time ventilation only may require 24-h ventilation during an acute illness for a short period of time. After sedation or anaesthesia, there should be increased monitoring and ventilatory support may be necessary even if the patient is awake. Ventilatory support is for lifetime, with no attempt to wean from ventilation.

During assisted ventilation, it is important to achieve normal oxygenation (SpO_2_ ≥ 95%) and a normal range for PCO_2_, ideally 4.6–6.0 kPa (35–45 mmHg). However, because the ventilatory requirements vary between sleep states, stable PCO_2_ levels are not always achievable. Some authors have suggested mild hyperventilation during sleep, e.g. PCO_2_ 4.0–4.6 kPa (30-35 mmHg), with the thought that this helped improve gas exchange the next day during spontaneous ventilation. But this approach has not been fully investigated in patients with CCHS and hyperventilation produces cerebral vasoconstriction.

Of importance, oxygen therapy alone improves oxygenation but may further aggravate hypoventilation, produce acidosis and result in coma.

### Ventilatory support requirements

Patients with CCHS have reduced tidal volume and respiratory rate during sleep and they often cannot generate adequate spontaneous breaths to trigger the ventilator.

***Pressure-controlled ventilation*** provides better ventilation than volume-controlled in case of variable or large leaks, e.g. with uncuffed tracheostomy cannulas or during mask ventilation. Only pressure-controlled mode on the ventilators secures adequate ventilation in CCHS patients at all ages with uncuffed tracheostomy cannulas or mask ventilation. Pressure-controlled ventilation (e.g. pressure control on the ventilators, or timed mode on the bi-level devices) is recommended. Inspiratory positive pressure and expiratory positive pressure are set independently to provide optimal tidal volume; respiratory rate and inspiratory time are set according to the patient’s age.

Newly-designed home ventilators have mixed ventilation modes, combining a pressure-controlled mode with a minimal guaranteed tidal volume or minute ventilation. Such devices may allow a reduction in the variability of PCO_2_ in different sleep states. Pressure support ventilation with a back-up rate, and minimum inspiratory time and safety guaranteed tidal/minute volume both appropriately set for age has been shown to be efficient in children of 4 years and older [47] and in one 10 month-old infant [78]. In a general setting, pressure support mode with no ability to set back-up rate and minimum inspiratory time on spontaneous breaths (e.g. pressure support on ventilators, spontaneous/timed -S/T- on some bi-level devices and spontaneous -S- modes on all bi-level devices) and continuous positive airway pressure (CPAP) mode should be avoided because they could lead to a variable breathing pattern.

***Home ventilators for positive pressure ventilation*** should be certified as safe, so they provide ventilation that is suitable for the age and weight of the patient (this is critical for infants and small children), have adequate visual and audible alarms (e.g. for disconnection, low and high pressures/volumes) and be equipped to provide sufficient power for several hours. For patients with tracheostomy, the ventilator should be certified for invasive use. Ventilators should allow maximum mobility, so they must be small sized and light for portability, have an internal battery, an external battery and connection to the car battery. Ventilators should be easy to operate, allowing parents or carers to change the settings if so prescribed. Commonly, ventilators have programmes with different settings for different conditions, e.g. wet and dry circuits, day and night time, well and sick settings.

Ventilators must be quiet or even silent in normal operation, so as not to disturb the patient and the rest of the family. Brands and models of positive pressure ventilators used at home vary between centres and countries, and regularly change, reflecting technical advances. Patients with CCHS should have a second, back-up ventilator available, or if not possible, there should be 24-h access to technicians that provide a replacement ventilator at short notice.

***Respiratory monitoring at home*** needs to be taken to avoid hypo/hyperventilation. Overnight monitoring using pulse oximetry with alarms is recommended in all patients, and periodic recordings of end-tidal or transcutaneous PCO_2_ should be obtained. The availability of reliable medical equipment for home use is a concern to patients and families. Transthoracic impedance monitors are not recommended because they fail to detect hypoventilation or obstructed apnoeas. Continuous nocturnal observation by trained carers is available in some countries, especially during mask ventilation.

### There are 4 types of ventilatory support

(i) Positive pressure ventilation via tracheostomy, (ii) Positive pressure ventilation via mask (mask ventilation), (iii) Phrenic nerve pacing, (iv) and Negative pressure ventilation. The choice of type and changes from one to another are influenced by a number of factors, such as the patient’s age, age of disease onset, duration of ventilator dependency, medical and centre experience of each technique, and the patient’s and families’ wishes. The decision should be individualised for each patient and take into account the benefits versus risks of each type, along with local availability. Some patients may experience different types of ventilatory support at the same time (e.g. on respiratory pacing during daytime and on ventilator during night time) or at different ages (e.g. transition from tracheostomy ventilation to mask ventilation) (Table 3).

**Table 3:** Benefits and risks of different types of ventilation support in CCHS

	Tracheostomy Ventilation	Mask Ventilation	Phrenic Nerve Pacing	Negative Pressure Ventilation
**Benefits**	• Provides effective ventilation at baseline and during infections • The airway is secured • Easy connection and disconnection • Enables prolonged continuous mechanical ventilation • Decreases dead space and airway resistance • Facilitates suctioning of secretions • Prevents obstructive apnoeas • Uses portable, battery-operated ventilators (favours mobility)	• Non invasive ventilation • Easy handling • Short training, facilitating discharge home • Avoids tracheostomy • Uses portable, battery-operated ventilators (favours mobility) • Reduces stigmatisation, better self-image • Allows speech development	• Enables mobility during ventilation • Psychological well-being as the patient is independent and the breathing is “more physiological” breathing. • Avoids mask-related facial deformation • Possibility of decannulation	• Non invasive ventilation • Avoids tracheostomy • Easy handling • Short training, facilitating discharge. • If used just overnight, the patient is device-free during the day • Costs less than tracheostomy • The face is free • Avoids mask-related facial deformation
**Risks**	• Invasive ventilation • Requires specialised care and long training • Daily tracheostomy care • Increases costs • May interfere with feeding • Risk of phonation & speech development delays • Potential stigma • Complications: infections, accidental decannulation, obstruction, granuloma, tracheomalacia, tracheo-cutaneous fistula after decannulation	• The airway is not secured • Potential ineffective ventilation due to leakage, upper airway obstruction or asynchrony • More limited interfaces in infants and younger children • Difficult to be used more than 18 h a day • Risk of facial deformation due to prolonged use • Potential discomfort, pressure sores, pain and non-cooperation that increase risk of self-disconnection • Potential for aspiration • Needs high degree of carer surveillance during sleep • May require temporary intubation when with infection or other challenges	• Surgical procedure for implanting electrodes and receiver • Requires highly-experienced medical centres • Can cause OSA requiring mask ventilation, when decannulated • May require alternative or additional form of ventilation support during illness • Pacer malfunction • Surgery is needed for replacement of electrode or receiver • The airway is not secured • There is no alarm to alert carers to antenna-receiver uncoupling in some devices • Not indicated under 1 yo • Pacing > 12–16 h/day is not recommended	• Ventilation is less effective • Lack of portability • The patient is not accessible for assessment and care (according to devices) • Can cause OSA requiring mask ventilation • The airway is not secured • Can cause aspiration in patients with swallowing disorders • Can cause back/shoulders pain and difficulty sleeping related to supine positioning • Can cause skin irritation and a sense of feeling chilled • May require alternative or additional form of ventilation support during illness

### Positive pressure ventilation (PPV) via tracheostomy

may be used in CCHS at all ages. This is the most commonly used type when considering all patients with CCHS. It has been recommended in neonates and young children with CCHS by the common assumption that this achieves better gas exchange and neurocognitive development during the first years of life. Only home ventilators with all the safety requirements are recommended for tracheostomy ventilation.

Tracheostomy is performed as soon as technically possible after diagnosis in newborns and young infants. Uncuffed cannulas are recommended in infants and children in order to reduce the risk of tracheomalacia and to facilitate speech with a speaking valve or a plug. Cuffed cannulas may be used in children for limited periods, such as during severe lower respiratory infections due to reduced lung compliance. The cannula size should be adjusted as the child grows in order to provide adequate mechanical ventilation at baseline and also during intercurrent pulmonary infections. Generally, smaller cannulas are preferred provided adequate ventilation is maintained. Adult patients are more likely to use cuffed cannulas in order to prevent leaks during assisted ventilation.

It has been advocated that there should be surveillance bronchoscopy to allow early diagnosis of granulomas and to assess cannula size and position. However, the periodicity has not been established [34, 74, 106]. We recommend that bronchoscopy should be considered in patients with CCHS for the following indications: (i) before decannulation; (ii) with symptoms, such as bleeding from the airway, breath holding spells, pain, cyanosis or desaturation during cannula changes, wheezing, recurrent infections, intolerance to a speaking valve or plugged tracheostomy cannula, change in voice quality; (iii) infants and young children in the first 2 years after creation of the tracheostomy, (iv) after changing the cannula size or type, to check its position using simple fibre-optic tracheoscopy.

### Mask ventilation

requires that the patient is cooperative, has a normal airway and needs ventilator support during sleep only (or has phrenic nerve pacing during the daytime). This is the first option in children and adults presenting with late-onset CCHS and requiring night time ventilation only. For patients who require daytime ventilatory support, mask ventilation should not be used alone, but may be considered in association with a respiratory pacing during wakefulness. There are infants with neonatal presentation of CCHS who have received mask ventilation without ever having had tracheotomy [71]. There is no available evidence comparing outcomes of children using tracheostomy ventilation and those with early mask ventilation. Mask ventilation needs a high degree of supervision by the carers during sleep, especially for infants and young children as there may be serious consequences of dislocation of the mask or obstruction of the upper airway.

Transition from tracheostomy to mask ventilation may be considered at varying ages, mainly for those with adequate ventilation during wakefulness [66]. Some authors consider the optimal time for transition as being older children (e.g. 10 years and older), but some children are co-operative and benefit at younger ages (e.g. 5–8 years) and before school entry. Procedure for transition may vary in different centres. Before decannulation, it can be helpful to downsize the tracheostomy cannula. Inspection of the trachea using a fiberscope by an ear, nose and throat (ENT) surgeon and sleep study while on mask ventilation with capped tracheostomy can be performed to assess feasibility of transitioning to mask ventilation. Close monitoring is made after decannulation [77].

Ventilation can be delivered via nasal or oro-nasal masks, nasal prongs or total face mask. Recent improvements in mask development and production have contributed to using mask ventilation as a first choice even in small infants. Total face mask has been discouraged by some because of patient discomfort and concerns for vomiting and aspiration, but it has been used to reduce pressure on facial structure or to prevent oral air leaks. Close maxillo-facial follow up is recommended for all patients with mask ventilation. Mask ventilation has been related to mid-face deformation, especially when introduced in the first years of life or with prolonged or tight application of the mask [75]. The severity of this complication may be reduced with newer total face masks, or by alternating between different shapes of masks and avoiding tight fitting.

Home ventilators are recommended for mask ventilation. Not all of the bi-level devices meet all the safety requirements on ventilators.

### Respiratory pacing

#### How does respiratory pacing work?

The most frequently used is phrenic nerve pacing. The pacing system consists of bilateral electrodes sutured surgically under the phrenic nerves, lead wires from the electrodes to subcutaneous radio receivers, and an external battery-powered transmitter with two coiled antennas. The external transmitter sends radio waves to the receiver implants. The receivers convert the radio waves into stimulating pulses that are delivered to phrenic nerves by the electrode [64, 82].

Inspiration is induced by repetitive stimulation of the phrenic nerve causing contraction of the diaphragm. When stimulation stops, passive expiration follows. This type of ventilation more closely resembles physiological breathing, as inspiration is induced by negative pressure within the chest. While unilateral pacing has been effective in adults, the paediatric population usually requires bilateral pacing to ensure adequate ventilation. Two phrenic nerve pacing systems are currently commercially available: a monopolar electrode system from Avery® Biomedical Devices Inc. (New York, USA), and a quadripolar electrode system from Atrotech® Ltd. (Tampere, Finland).

#### Indications, benefits and risks

Phrenic nerve pacing requires functional phrenic nerves and diaphragms. This can be assessed by using observation of the diaphragm movements during voluntary deep breathing using sonography or fluoroscopy or transcutaneous stimulation of the phrenic nerve at the neck together with sonography of the diaphragm [17, 89]. Phrenic nerve pacing is considered as a mode of daytime ventilatory support in patients with CCHS needing > 16 h/day ventilation and at least older than 1 year of age. Relative contraindications to respiratory pacing include chronic lung or airway disease, including obstructive sleep apnoeas, severe behavioural disorders and obesity (Diep 2015, [86]).

In patients under 6 years of age, pacing with a tracheostomy provides a greater stability of tidal volume, oxygen saturation and PCO_2_ than pacing without tracheostomy. Closure of the tracheostomy in this young group will often be complicated by recurrent obstructive apnoeas during sleep. Between 6 and 12 years old, successful decannulation is feasible [105], but should be done with careful observation for obstructive apnoeas [98]. Before decannulation, it can be helpful to downsize the tracheostomy for a couple of weeks and to perform sleep studies with tracheostomy capped while the patient is ventilated by phrenic pacing in order to assess obstructive sleep apnoea and modify pacing settings to optimize ventilation [21, 26, 101]. Problems with upper airway obstruction, e.g. after decannulation and closing the tracheostomy, can be managed by increasing the inspiratory time and decreasing the force of inspiration. If present, carers can also re-position the head or neck to lessen the occurrence of obstructive apnoeas. Mask ventilation may be needed as an ultimate option.

The benefit of phrenic nerve pacing is greatest in patients, who need ventilator support 12–24 h/day. In these patients, the pacemaker offers freedom from the ventilator during the day. They use the small, battery-operated, and easily portable pacing transmitter during the day, allowing greater mobility and participation in sporting activities. During the night, patients should use a positive pressure ventilator. Pacing more than 12–16 h/day is generally not recommended.

The risk of post-surgical implant infection is about 6% and requires surgical removal and replacement of the implants. The incidence of mechanical lesion to the nerve differs between centres; overall it is about 2%. In most cases, phrenic paralysis is transient [21, 104].

Pacer malfunction on one side is mostly caused by a defect of the antenna, and new spare antennae should always be available at home. A defect of the transmitter is rarer, and a backup transmitter should also be available. After years of pacing, a defect in the implants can occur, most frequently due to a break or an insulation defect of the wire between receiver and electrode. The receiver can also be damaged from external mechanical trauma. A rare cause of pacemaker failure includes wire breakage from children’s repetitive handling of the receivers [30]. Defects of the receiver need subcutaneous replacement of a new receiver, which is relatively straightforward and can result in resumption of pacing in 1–2 days [104]. Defects of the electrodes or their wires need thoracic surgery, which may mean a delay of 2–6 weeks before restarting pacing.

#### Technical procedure

A surgical procedure is required for implanting electrodes and receivers. While some prefer a cervical approach, a majority of centres adopts intrathoracic implantation of the phrenic nerve electrodes [64]. The latter can be done by open thoracotomy under general anaesthesia or by less invasive thoracoscopic techniques. The receivers are implanted subcutaneously on the lower portion of the thorax or on the abdomen immediately below the 12th rib and connected to the electrode wires.

Regular pacing is started not before 10–14 days after implanting the electrodes, in order to allow wound healing and resorption of post-operative oedema. Some authors recommend waiting 4–6 weeks before starting to pace, as this allows any tissue reaction around the electrodes to stabilize [82]. Pacing is initiated and controlled with medical monitoring providing the appropriate settings and training the patient. The duration of first pacing should not last longer than 1–2 h/day and increases weekly by 30–60 min/day. Thus, a training period of 2–4 months is usually required to achieve full pacing of 12–16 h/day. Phrenic nerve pacing requires careful training of the caregivers and the patients, and at least yearly follow-up in a centre with expertise in phrenic pacing. The aim is “to minimize electrical stimulation to the phrenic nerves and yet achieve adequate ventilation and oxygenation”.

#### Follow-up

Successful pacing depends on both surgical expertise and pacing expertise, e.g. for adjusting settings and dealing with pacer malfunction. The first in-hospital evaluation should be done 6 months after implantation, and then annual in-hospital checks are recommended. It is important that there is continuous monitoring of SpO_2_ and PCO_2_ during sleep and daytime activities during a 24-h period, so that settings may be adjusted. Additional monitoring includes ECG during pacing: analysis for inspiration time, pulse interval and evaluation of the diaphragmatic muscle potentials; and in case of pacer malfunction, phrenic nerve conduction studies.

### Negative pressure ventilation (NPV)

The negative pressure ventilator consists of a rigid cuirass, wrap or tank ventilator that encloses part of the patient’s body below the neck. An attached pump produces a negative pressure in the device that results in expansion of the ribcage and thus promotes inspiration. Use of NPV is now limited because of its lack of effectiveness due to air leaks around the cuirass and to upper airway obstruction, its reduced portability, its inability to be battery operated, and difficulties in sleeping in the supine position and skin irritation. There are rare cases of CCHS currently using NPV, most from England and Germany. One currently available device is the RTX® cuirass (Hayek Medical®).

## Management of Associated Disorders

### Cardiovascular disorders

The cardiovascular problems include: i) cardiac arrhythmias due to autonomic dysfunction, including sinus node dysfunction, sinus pauses and sinus bradycardia, reduced heart rate variability, reduced heart rate response to exercise, and vasovagal syncope; the occurrence of prolonged R-R intervals in CCHS patients has been proposed as a risk for sudden death [36], and ii) blood pressure (BP) dysregulation that leads to high levels of BP at night and arterial hypotension during the day, postural hypotension [91]. Patients may manifest recurrent episodes of dizziness or fainting.

In all patients with CCHS, an annual routine ECG Holter of 48–72 h is advisable. No evidence is available on the minimal duration of ECG Holter recording. In patients with CCHS presenting with syncope or fainting, one should exclude seizures, breath-holding episodes, hypoglycaemia, postural hypotension or other illness. If no cause is found, ECG Holter recording should be longer or repeated e.g. 3–6 months or patients should have an implantable loop recorder inserted [60]. Patients with syncope occurring without the finding of bradycardia or long sinus pauses have still sometimes undergone implantation of a cardiac pacemaker [51].

One single-centre study has reported presence of R-R intervals ≥3 s in a higher proportion in patients with 20/27 genotype than in those with 20/26 or 20/25, suggesting a relationship between PHOX2B genotype and the prevalence of sinus node dysfunction [36]. However, this relationship is variable and recent data suggested that all individuals with CCHS should be screened for sinus pauses [51].

Current recommendations for cardiac pacing in sinus node dysfunction are published for general cohorts (without CCHS) by task forces from different scientific societies [17, 50]. The main indication for cardiac pacing is a serious clinical symptom (e.g. syncope) occurring in patients with proven profound bradycardia or long sinus pauses. Considering the lack of data regarding patients with CCHS, we recommend using the ACC/AHA or HRS guidelines for pacemaker implantation [17, 50]. These advise that symptomatic patients with recurrent syncopes and R-R intervals ≥3 s should undergo implantation of a cardiac pacemaker.

In CCHS patients without symptoms, but with R-R intervals of ≥3 s, an individual decision must be made based on age, frequency of sinus pauses, length of longest R-R intervals and symptoms. For example, in an infant without symptoms, but with R-R intervals of ≥3 s, a decision for cardiac pacemaker implantation seems reasonable. Conversely, a young adult presenting with longest R-R interval of 3.1 s duration, 20/25 PHOX2B genotype and no symptoms may be considered for observation with regular scheduled ECG Holters or an implanted loop recording.

In patients with proven exclusive sinus node dysfunction, a single chamber atrial pacing device (AAI-mode) may be sufficient. However, a dual chamber pacemaker (DDD-mode) is preferable to ensure cardiac stimulation in case of subsequent development of atrioventricular block and for hemodynamic advantages in the case of vasovagal syncopes [49]. To avoid interference with a phrenic nerve pacer, the cardiac pacer should have a bipolar electrode.

### Digestive disorders of lower and upper gastro-intestinal (G-I) tracts

***Hirschsprung disease*** (HD) occurs in about 13–20% of patients with CCHS. This disorder is caused by an absence of ganglion cells from the neural crests-derived enteric nervous system, predominantly in the distal colon. The affected gut segment can vary from short, restricted to the rectum and sigmoid colon, to long segment, which can extend to the more proximal colon. Presence of HD is a risk factor for increased morbidity symptoms depending on the length of the affected gut.

Most patients are diagnosed in the first months of life, neonates failing to pass meconium in the first 24 h, while older infants can develop abdominal distension, vomiting, diarrhoea, and constipation. Constipation that responds poorly to medical management is a major symptom. Constipation may exist without HD, reflecting large bowel dysmotility.

HD should be investigated in any patient with CCHS presenting with suggestive symptoms, regardless of age or severity of symptoms. We do not recommend systematic investigation in asymptomatic patients. The gold standard for diagnosing HD is rectal suction biopsy. If an adequate specimen is obtained, sub mucosal ganglion cells are not identified and acetyl cholinesterase activity is elevated [1]. Anorectal manometry is a highly sensitive test that assesses the recto-anal inhibition reflex, which is absent in HD. Contrast enema is the least sensitive and specific of all three methods especially in the first weeks of life. Definitive treatment for HD is surgical removal of the affected gut and dietary adjustments [18].

***Affected upper GI tract*** mainly results in (i) oesophageal dysmotility with swallowing difficulties, especially with solids, vomiting or can be asymptomatic and (ii) gastro-oesophageal reflux (GER) which favours recurrent vomiting, chronic nocturnal cough and retrosternal pain [28].

Oesophageal dysmotility can be identified by upper GI contrast studies and oesophageal manometry even in asymptomatic patients [28]. In infants and toddlers with GER, endoscopy findings of mucosal breaks and inflammation are reliable evidence of reflux; pH monitoring shows oesophageal acid exposure, but its clinical utility for diagnosing or management of gastro-oesophageal reflux disease is less well established. Contrast studies are not reliable for the diagnosis of GER.

Effective medications are histamine-2 receptor antagonists and proton pump inhibitors. In refractory, chronic, relapsing GER disease, anti-reflux surgery is indicated. It is helpful to have advice from speech and language therapists.

### Ocular disorders are

found in 46–92% of patients with CCHS [35]. The commonest are pupillary abnormalities, convergence insufficiency, strabismus or ptosis. They can be categorised into three types of disorders:

(i) ***Intrinsic ocular anomalies*** include various pupillary defects, and abnormal pupillary light responses with poor dilation. Bilateral severe miosis is observed much more commonly than mydriasis, while the latter can occur with third cranial nerve palsy. Anisocoria is a common finding, but more in association with miotic pupils than mydriatic pupils. Pupillary hippus with unstable pupils is a clinical sign at biomicroscope examination;

(ii) ***Extrinsic oculomotor anomalies*** include various forms of convergence insufficiency (exophoria, esophoria) with variable angles. Different features of strabismus, including exotropia or esotropia, can also be found. Specific types of strabismus, such as Brown syndrome, have been described. Cases of complete or partial third cranial nerve palsy may be observed, but isolated ptosis may be part of a third cranial nerve palsy or a congenital form involving innervation anomalies.

(iii) ***Ocular globe disorders*** may be related to CCHS or be incidental associations. Morphologically abnormal irises with smooth iris and absence of crypts or transillumination defects have been detected. More severe manifestations, such as microphthalmia, have been observed. Lachrymal obstruction is more likely from chance association, being a common pathology in children. Insufficient production of tears has been found and often associated with hemi-facial sweating. No specific association with lens or retinal disorders has been noted in CCHS.

A complete ocular examination should be performed at diagnosis of CCHS, then a review scheduled annually or more frequently if ocular disorders are recognised. Symptoms, such as headaches, visual difficulties, or strabismus, should prompt ocular examination. Some children may suffer obvious clinical symptoms, such as strabismus, ptosis or third cranial nerve, palsy but some pathology needs specific ophthalmology review before it can be treated. The visual system is not mature at birth so the ocular signs must be evaluated taking into account the age of the infant or child. For example, intermittent strabismus might be physiological during the first 3 months of age.

Ocular motility is assessed with corneal reflex, red reflex and cover test. Visual acuity in verbal children, anterior segment evaluation with biomicroscope and fundal examination are systematically performed, including full pupillary dilation. Refraction with cycloplegic examination should also be completed by an orthoptist to define binocular vision, especially if asthenopia is an alleged symptom. Stereoscopic tests should be performed. In infants, visual acuity and cycloplegic examination are unnecessary, but hand-held biomicroscope and automated refractometer are useful.

Refraction errors can receive optical correction, while some binocular disorders involve re-education. Strabismus and ptosis correction may require surgical procedures. Early treatment has a better outcome, but first needs recognition. Specific attention is necessary to detect convergence insufficiency and prevent amblyopia to minimise learning problems.

### Neurological disorders

Life-threatening neurological complications may include: (i) breath-holding spells in infants and toddlers, often triggered by anger, fear or pain. Some may result in profound desaturation and/or bradycardia, followed by loss of consciousness. Tracheo- or bronchomalacia should be investigated in case of recurrent episodes. (ii) seizures are mostly secondary to severe hypoxaemia, hypoglycaemia, or arrhythmia. (iii) syncopes or fainting episodes may occur in up to 25% of patients with CCHS. They may be caused by sinus bradycardia or transient asystoles or postural hypotension.

Neuro-developmental outcome is of increased concern in patients with CCHS [16, 56, 76, 85, 107]. Overall, the findings showed a wide range in overall intellectual functioning from normal to moderate IQ, suggesting some impairment of intellectual functioning in CCHS. Impairment in visual-perceptive skills, attention, language, memory, learning and school performance has been noted. Patients with CCHS may have attention and concentration problems and approximately 66% of children obtained a score below the borderline cut-off in the visual/auditory memory skills. Parents reported that 30% of subjects had a formal diagnosis of learning disabilities. Half of the children with CCHS had additional educational needs or were in special schools. The quality of ventilation and oxygenation in the first years of life are of importance for the neurodevelopment.

Individuals with CCHS may have reduced anxiety [69], and 54% of the patients with CCHS showed difficulties in social interactions [61]. Furthermore, parents reported adaptive function problems affecting communication and daily living skills. These findings are unsurprising given that social stigmata associated with medical care represent an additional risk factor for interpersonal relationships and the participation in social activities.

Psychomotor development should be assessed in the first year of life. Comprehensive neurocognitive follow-up should be repeated at least annually in the first few years, in order to plan for special educational needs and/or psychological support. Intensive educational intervention allied to careful respiratory management is very important in order to maximize the child’s full neurocognitive potential.

### Metabolic/endocrine disorders

Glycemic regulation is under autonomic control. Abnormal hypoglycaemia, commonly associated with hyperinsulinemia, has been reported in patients with CCHS [33, 38, 40, 57] and can manifest with seizures. Hypoglycaemia can be asymptomatic, but when there have been seizures, requires dietary adjustments with or without diazoxide [62]. Hyperglycaemia has also been documented. Growth hormone deficiency has been reported in one patient [92, 93] and hyperthyroidism in another [31].

### Neural crest-derived tumours

These tumours may occur in 3–5% of patients with CCHS. Neuroblastomas, ganglioblastomas, and ganglioneuromas are at high prevalence in PARMs with longer expansions and in non-PARMs [37]***.*** They are often found within the neck, chest and abdomen. Symptoms may depend on type and localisation of the tumour: specific search may include clinical examination, chest x-ray, abdominal ultrasound and MRI and MIBG scan. In these cases, a more intensive approach to treatment may be taken. The type and frequency of assessment is guided by local oncology protocols.

## Living with CCHS

### Communication of diagnosis

A newly diagnosed patient and his family should be directed to a centre with expertise in the management of CCHS. This is of particular need for such a rare disease. A face-to-face empathic communication should take place in comfortable surroundings with the utmost privacy. Patients (or parents) should be educated and empowered to care whilst they remain in hospital and be engaged early in discharge planning. Genetic counselling is required, with regard to the genetic transmission and possible risk of recurrence. The mutual help offered by family associations represents an invaluable resource for patients (or parents).

### Standards of home care

Patients with CCHS are technology-dependent, but aim to live at home, engaging in activities as normally as possible. Patients (or parents) should receive extensive training in CPR, airway management and equipment handling. Practical skills should reach the standards of nurses/carers caring for such patients. Early discharge planning from hospital can be greatly facilitated by ensuring a case manager, such as a clinical specialist nurse or lead community nurse.

***(i) Patient-related issues*** and emergency situations should be fully characterised. Of particular importance is the identification of possible episodes of hypoventilation during wakefulness and the management of equipment failure.

***(ii) Home equipment*** should be available in a dedicated room where the patient sleeps. The basic equipment consists of a portable home ventilator, a suction pump, a pulse oximeter and a self-inflating bag. In addition, spare tracheal cannulas, suction catheters, syringes, saline etc. are needed for daily use. A second back-up ventilator must be available. Oxygen may be recommended for emergency use at home or when out of home. A PCO_2_ monitor can help to detect hypercapnia and to avoid inadvertent hyperventilation. Mobility is supported by an additional portable pulse oximeter and suction pump. Toddlers or young children need a stroller large enough to accommodate the ventilator and all equipment.

***(iii) Family members and professional caregivers*** should be trained for care of CCHS outside the hospital. There should be availability of at least two trained care givers, serviced equipment devices and consumables, a schedule for follow up, care from a primary physician, and supervision from a specialist nurse. Specific educational programs for carers are mandatory for optimal care of patients with CCHS. Particular attention should be dedicated to young children with tracheostomy ventilation, even more in single parent families. Support by nurses and carers competent to meet the health care needs of the child, at least overnight or for several hours per day, to allow parents sleep or to attend to other children. While this is not allowed in some countries, some health care systems provide “ventilation ladders”, which provide criteria for when parents can adjust ventilator settings before calling for medical assistance, e.g. when SpO_2_ falls below prescribed limits.

***(iv) Organisational issues*** should have been solved. Families must have equipment service contract in place, and ideally 24-h telephone access with a reference centre for CCHS Families. Checklists with all emergency telephone numbers can be helpful. A primary physician must also be involved in routine patient care.

### Follow-up

Patients should be followed up in a reference centre that at least regularly manages patients with CCHS, regularly manages home ventilation, has regular access to intensive care in the hospital and regularly performs cardio respiratory recordings (with PCO_2_) (Additional file 1). In addition, the centre should be able to diagnose CCHS, provide regular multidisciplinary reviews and take part in the registry and research activities. Genetic testing and implementing respiratory pacing can be contracted with specific units.

Care for paediatric patients is to be coordinated by paediatricians, intensivists and pulmonologists. The team includes experienced physicians, specialised nurses/respiratory technicians, physiotherapists, social workers and psychologists. Staff in hospitals and care providers at home should be competent to adjust treatment to each patient’s needs. Because of the rarity of CCHS in adults, it is recommended that adult patients are managed in collaboration with a reference centre.

A follow up programme is recommended for regular check-ups by physicians with knowledge of CCHS. Children younger than 2 years of age need more frequent respiratory review (e.g. every 4–6 months), including review of the tracheostomy or specific maxillo-facial follow up for those on mask ventilation. A sleep study (full night polysomnography or cardio-respiratory recording) with oximetry and PCO_2_ is recommended regularly to detect hypo- or hyper ventilation, patient-ventilator asynchrony and sleep quality. A comprehensive assessment of cardiac, digestive, and ocular dysautonomia should be made regularly. Neurodevelopmental assessment using age-adapted tests is needed at least annually thereafter until school age and throughout childhood and adolescence for educational needs assessments. Patients carrying longer PARMs or NPARMs need regularly screening for neural crest tumours, especially during childhood (Table 2).

After the first hospital discharge, there should be close follow up by a local pulmonologist or paediatrician to ensure a smooth and safe transition from hospital to home care. Infants and young children should be reviewed every 1–2 months in the first year, and every 3–4 months in the second year. There should also be review at the reference centre at least annually thereafter (Table 2).

### Emergencies

Urgent hospitalisation is needed in the case of acute respiratory deterioration (such as cardiorespiratory arrest, severe hypoxaemia, inability to manage accidental decannulation, bleeding from the stoma, etc), any alteration of consciousness, or syncope. Carers should be able to identify critical situations rapidly so that they can take remedial action before hospital admission. The emergency medical services should be informed of the patient’s condition and ventilatory support needs.

### Daily life

Mobility is encouraged. Parents and carers must be familiar with procedures for care at home and also during outdoor activities. Portable battery-operated ventilators allow ventilation during naps in infants with CCHS. Moving from tracheostomy to mask ventilation permits reduced daily care. Phrenic nerve pacing should be considered in patients dependent on ventilation during the daytime.

Normal schooling is possible for children with CCHS although some of them may have learning difficulties and require special teachers or therapists. Educators should be informed of the child’s condition and be prepared to act in case of any event. In addition, children may be entitled to receive medically related services by a nurse, especially in case of a tracheotomised patient. Educational needs should be reviewed annually.

Sleep is disrupted in children with CCHS and their parents [65] and warrants professional support i.e. by trained nurses, although this is not universally available.

Sports that involve moderate exercise are encouraged, allowing frequent breaks due to the potential inability to increase ventilation in response to increased metabolic demand. Strenuous activity may be a risk, especially after the end of exercise as the respiratory compensation of tissue acidosis may be less effective and lead to autonomic dysfunction. Swimming should only be permitted under close surveillance, controlling and limiting the immersion time because patients with CCHS have no perception of hypoxia-related dyspnoea and may become profoundly hypoxic, lose consciousness and drown. Free diving and underwater activities are strongly discouraged.

Air travel is possible for patients with CCHS. Of note, commercial aircraft flight is associated with significant altitude exposure, so the inspired fraction of oxygen in the cabin is equivalent to that at about 2700 m (similar to 15% at sea level). Patients with CCHS fail to increase ventilation in response to hypoxia and cannot receive additional oxygen alone without assisted ventilation. We advise that a ventilator is available for use in the cabin if SpO_2_ falls in flight to less than 90%. While this may not restore normal SpO2 levels, oxygen should be added cautiously. Support from the physician should be sought in advance of any intended flight, so a hypoxic challenge test, also known as a Fitness-to-Fly test and liaison with the airline can be discussed. Appropriate carry bags for equipment and the correct electrical adapters for the destination countries are important practical issues.

Independence is encouraged for young adults with CCHS although they may have reduced or absent perception of audio alarms of the ventilators while asleep. Ultimately, some remain with parents/family while others find partners who learn to care, or live in communities for young adults with varying disabilities, or live alone with a carer visiting at night, or have dogs trained to respond to monitor alarms. While varied, measurements of the quality of life for young adults appear to be only moderately impaired [100].

Pregnancy of patients with PHOX2B mutation-positive CCHS should be planned only after genetic counselling. They are informed about the risk of recurrence and the available methods of ante-natal diagnosis or preimplantation genetic diagnosis (PGD). Ante-natal diagnosis is advised and while this may not be performed for the purposes of determining whether to continue with the pregnancy, it can help early post-natal planning and intervention. During pregnancy, labour and delivery, pregnant women with CCHS often require increasing ventilatory support secondary to elevated abdominal pressure. After caesarean section, phrenic nerve pacing is painful, and should be replaced by mask ventilation for a few days. Affected foetuses should be born in a centre able to initiate ventilation and care in collaboration with a reference centre for CCHS.

Anaesthesia is a challenging situation for patients with CCHS as most anaesthetic agents further depress ventilation and enhance autonomic dysfunction [11, 13]. Direct supervision by anaesthetic staff, ventilatory support, as well as blood gases, blood pressure and glycemic monitoring should be maintained throughout the process before, during and after surgery and anaesthesia. Short acting agents, volatile agents like sevoflurane [42] or regional anaesthesia if possible [90] are preferred. Of note, propofol is now widely and safely used during short procedures and anaesthetic induction in patients with CCHS [13, 63], although one case has been report of heart block occurring after propofol bolus at induction of anaesthesia in a young child boy with CCHS [87]. Preoperative and postoperative ventilation and non-invasive blood gases monitoring are recommended, even when the patient is awake. This especially applies to patients in need of opioid-based pain management [90]. Of note, a prolonged need for mechanical ventilation after sedation or anaesthesia may be the first presentation of CCHS with mild phenotypes [54, 55].

Some everyday medications (anti-cough, anti-pain, sleeping pills) may contain agents with sedative, opioid actions that may further depress ventilation in patients with CCHS even while awake. Alcohol and many illicit substances are known as respiratory depressants. Parents and patients, particularly teenagers should be alerted to the adverse effects associated with alcohol consumption, including risks of coma and death [21].

## Research questions

- Improving knowledge of PHOX2B biology: what are its target genes, how its expression is regulated, when and in which cells it functions and to do what, etc.
- Improving the sensitivity of genetic tests for mosaic carriers
- Understanding the molecular basis of reduced penetrance and variable expressivity of PHOX2B mutations
- Improving knowledge of physiopathology underlying deficiency of chemosensitivity
- Identifying a drug to sufficiently enhance spontaneous ventilation in patients with CCHS
- Improve knowledge of genotype-phenotype relationship
- Technological advances in monitoring and ventilation e.g. development of a SpO_2_-monitor to ventilator (or phrenic pacer) feedback system ensuring stable blood gas levels
- How to help young adults become more independent
- Development of tools to assess quality of life in different age subsets of patients with CCHS
- Methods for identifying the causes of developmental delay, including tools for learning more about long term oxygenation and sleep quality
- Understand the lifelong trends in hypoventilation and the complications that arise during adulthood
- To collect data on long term facial growth and relate that to modality of ventilation
- Role of continuous monitoring of SpO_2_ on long term outcome
- Hypoxic challenge tests in CCHS / safety with air travel
- Interest of telemedicine for both video-consultation and in cardiorespiratory monitoring

## Conclusion

CCHS is a rare disease, affecting newborns, children, adults and families. Knowledge of the disease continues to evolve in the last decade, with regard to the clinical presentation, genetic support, and management. Table 4 outlines the main differences observed within this time period (i.e., the current guidelines versus the 2010 American Thoracic Society (ATS) statement [106]. Management of CCHS requires lifelong ventilatory supports and multi-specialty follow-up, the quality of which garantees the longterm clinical outcomes. Awareness and knowledge of the disease are to be brougth to a large health community including adult physicians and health social workers.

**Table 4:** Main differences between the current guidelines and the 2010 ATS statement

I**ssue**	C**urrent** G**uidelines** (2020)	ATS S**tatement** (2010)
**Phenotype**	Disease may have varying respiratory impact, with predominance of other system dysfunction	Hypoventilation with other autonomic disturbance
**Cardiac issues**	All CCHS at risk for sinus arrest	Longer PARMs at risk for sinus arrest
**Targets for ventilatory support**	pCO_2_ 35–45 mmHg SpO_2_ ≥ 95%	Reasonably: Et CO_2_ 30–50 mmHg Ideally: PCO_2_ 35–40 mmHg SpO_2_ ≥ 95%
**Ventilation support**	Use of new modalities, such as volume guaranteed to allow varying needs to be met with less swings in CO_2_	Largely non-varying tracheostomy / mask ventilation
**Tracheostomy ventilation**	The commonest method of ventilation support in the first years of life	Recommended in the first years of life
**Airway assessment procedure**	Simple fibre-optic tracheoscopy preferred	Bronchoscopy
**Indications for Airway assessment procedure**	-If new symptoms -After changing tube size or type -Before decannulation -Every 3–6 months in children in the first 2 years after tracheostomy	Every 12–24 months
**Ventilatory Device**	-Home ventilators while on tracheostomy and mask ventilation: -Home ventilators or bi-level devices with all safety requirements while on mask ventilation	-Home ventilators while on tracheostomy ventilation -Bi-level devices on timed mode while on mask
**Ventilation mode**	-Recommended: Pressure-control ventilation (e.g. pressure control on the ventilators, or timed mode on the bi-level devices) -To be avoided: Pressure support mode with no ability to set back-up rate and minimum inspiratory time on spontaneous breaths, and CPAP mode.	*-Recommended: Pressure control or pressure plateau mode* via *tracheostomy* *-Bi-level positive airway pressure ventilation by mask or nasal prongs: timed mode*
**Non-invasive ventilation** **(mask ventilation)**	-May be considered in infants and young children with close monitoring -The first option for older children and adults presenting with late-onset CCHS	-Not considered as an optimal mode of ventilation in infants and children -Not considered until 6–8 yo at the earliest in stable patients on sleep time ventilation only
**Prevention of mid-face hypoplasia (related to mask ventilation)**	-Use of total face masks -Alternating masks of different shapes	Extreme caution recommended while used in young children
**Mask models available**	Total face mask used to reduce pressure on facial structure or to prevent oral air leaks	Full face mask discouraged because of discomfort and aspiration risks.
**Age at transition from trach to mask ventilation**	Can be initiated at varying ages during childhood	After 6 to 8 yo
**Procedure for transition from trach to mask ventilation or phrenic nerve pacemaker**	-Tracheal fiberscope -Downsize the tracheostomy cannula -Sleep study while on mask ventilation or pacing with capped tracheostomy	/
**Is PHOX2B the sole CCHS gene?**	-A few other genes, responsible for autosomal recessive hypoventilation, like MYO1H and LBX1, were identified in consanguineous families negative for PHOX2B mutations. -CCHS is a genetically heterogeneous trait	Besides CCHS associated PHOX2B mutations, only patients carrying coincidental mutations in genes already involved in other neurocristopathies and/or in the development of Neural Crest derived cell lines were reported
**Novel kind of CCHS associated PHOX2B anomalies**	Interstitial deletions of the PHOX2B found in association with atypical CCHS presentations, neonatal respiratory distress, Hirschsprung disease, BRUE, etc	Unknown at that time
**Mutation penetrance and expressivity**	For most PARMs and NPARMs, a wide variability in intra-familial mutation penetrance & expressivity is emerging.	Reduced penetrance only reported for a few NPARMs and the shortest PARMs
**Genotype – phenotype correlation**	Differences for NPARM have been recognized both within and between missense, nonsense, and frameshift mutations	NPARM mutations did have roughly the same effect without distinguishing among them

## Statements

### Statements for diagnosis

- Any child with unexplained central hypoventilation should be investigated for CCHS
- Hypoventilation is typically more severe during sleep than wakefulness and during NREM sleep than during REM sleep in CCHS. Absent or low ventilatory responses to hypercapnia are present at both sleep and wake states.
- All newly-diagnosed patients and their families should be directed to a centre with expertise in CCHS.
- Mutations of PHOX2B are found in 90% of patients with CCHS
- Polyalanine expansion of PHOX2B is the commonest mutation found in CCHS
- According to the type of genetic test performed, either polyalanine expansions and nucleotide substitutions or PHOX2B interstitial deletions can be detected
- Once a PHOX2B variant is found in the proband, testing should be offered to parents
- Transmission of PHOX2B mutations is autosomal dominant with variable penetrance
- PHOX2B mutation have variable expressivity (in the presence of a same mutation, degree of severity may vary from patient to patient)
- Risk of recurrence can only be defined for CCHS with PHOX2B mutation
- Most patients’ parents do not carry the PHOX2B mutation found in their children
- Parents who carry the mutation may have all their cells mutated (constitutive carrier) or only a proportion of their cells mutated (mosaic carrier)
- Prenatal genetic testing is advisable in every family with a CCHS child, whether one of the parents is a carrier or not

### Statements for Ventilatory management

- Ventilatory support is life support in CCHS
- CCHS needs life-long ventilatory support
- Respiratory assessment during spontaneous and assisted ventilation while asleep and awake guides the decision making about ventilatory support for each individual patient. The adequacy of ventilation is estimated only on the basis of oxygenation AND carbon dioxide measurements
- Targets for ventilatory support are pCO_2_ 35-45 mmHg and SpO_2_ ≥ 95%
- Each ventilated patient at home needs to be monitored with oximetry and have access to end-tidal or transcutaneous PCO_2_ monitoring
- Highly trained carers are recommended, especially at night
- Home ventilators are recommended for tracheostomy or mask ventilation. Not all of the bi-level devices meet all the safety requirements present on ventilators.
- Pressure-controlled ventilation (e.g. pressure control on the ventilators, or timed mode on the bi-level devices) secures adequate ventilation in CCHS patients at all ages. Inspiratory positive pressure and expiratory positive pressure are set independently to provide optimal tidal volume; respiratory rate and inspiratory time are set according to the patient’s age.
- Pressure support ventilation with a back-up rate, minimum inspiratory time and safety guaranteed tidal/minute volume both appropriately set for age has been shown to be efficient in children older than 4 years.
- Pressure support mode (e.g. pressure support on ventilators and S/T on some bi-level devices, and S modes on all bi-level devices) and CPAP mode should be avoided because they lead to a variable breathing pattern
- Tracheostomy ventilation is the commonest method of ventilatory support in the first years of life
- Uncuffed cannulas are recommended in infants and children
- Supplemental warming and humidification of inspired gas is recommended during mechanical ventilation or even spontaneous ventilation through a tracheostomy cannula in small children.
- Bronchoscopy is recommended (i) before decannulation, (ii) for symptoms, (iii) in infants and small children in the first 2 years after tracheostomy inserted, and (iv) after changing the tube size or type, to check the position of the tube tip (with a simple fiber-optic tracheoscopy)
- Mask ventilation requires a cooperative patient with normal airway and either adequate spontaneous ventilation or phrenic nerve pacing during wakefulness
- Mask ventilation may be considered in infants and young children with close monitoring
- Mask ventilation is the first option for older children and adults presenting with late-onset CCHS
- Transition from tracheostomy to mask ventilation can be initiated at varying ages
- Mid-face deformation may be reduced by using well-fitting masks or alternating masks of different shapes or using total face masks
- Long-term follow up by maxillofacial and dental specialists is recommended
- Phrenic nerve pacing offers freedom from the ventilator during daytime in patients ventilated 24 h a day, thus increasing mobility and allowing sporting and professional activities

### Statements for associated disorders

- Prolonged asystoles are the most important disorder associated with syncope in CCHS
- Regular ECG Holter monitoring is recommended.
- In patients with syncope without a cause, ECG Holter recordings should be repeated more frequently, e.g. 3–6 monthly or an implantable loop recorder considered.
- Cardiac pacemaker implantation should be performed in patients with CCHS presenting with symptoms (e.g. syncope) together with profound bradycardia or long sinus pauses (Class I) according to international recommendations
- Cardiac pacemaker implantation should be considered in asymptomatic patients with life-threatening sinus pauses
- To avoid interference with a phrenic nerve pacer, the use of bipolar cardiac pacing electrode is preferred.
- Hirschsprung disease should be investigated in any patient with CCHS presenting with suggestive symptoms, regardless of age or severity of symptoms
- Swallowing difficulties and gastro-oesophageal reflux are likely frequent
- Ocular examination should be performed in all patients with CCHS
- Pupillary abnormalities, convergence insufficiency, strabismus and ptosis are the most common ocular disorders
- Optimal assessments include standardised psychometric instruments to evaluate mental and psychomotor development, cognitive and behavioural/psychosocial abilities, visual-perceptive skills, attention, language, memory, learning and school performance
- During breath holding spells, seizures or syncopes, one should ensure adequate ventilation and oxygenation.

### Statements on daily life

- Patients should have their own emergency/travel kit, including battery-operated devices
- Staff in hospitals and care providers at home should be competent in ventilatory support with monitoring adequate to the needs of CCHS patients
- Equipment should be maintained, including regular servicing, in line with manufacturer’s recommendations
- A thorough follow-up programme depending on the patient’s age and condition is of uppermost importance before discharge home
- Small equipment size should be used to optimise mobility
- Families should obtain approval from their physician and the airline before undertaking any flight. Health insurance to cover their travel should be obtained.
- Patients with CCHS are at increased risk of hypoxia and are unlikely to have a compensatory increase in minute ventilation during air travel
- Patients in flight should have ventilators or pacing transmitters, SpO_2_ monitoring and their travel kit in the cabin
- Usual recommendations apply to starting ventilation during air travel, but should also be considered if SpO_2_ falls to ≤90%, even during wakefulness
- If a cuffed tube is used, replace the air of the cuff with saline before boarding
- Non-invasive monitoring of blood gases and assisted ventilation are mandatory until complete recovery from anaesthesia
- Short procedures like sedation for dental surgery can be performed with short acting volatile anaesthetics
- Local anaesthesia should be considered to lessen the likelihood of systemic disturbance
- Preoperative evaluation must include comprehensive review of this multi-system disorder
- Sedative premedication must be performed only with the direct anaesthetic supervision and availability of monitoring and mechanical ventilation
- Intra-operative monitoring must be thorough, with invasive monitoring considered in specific situations
- Maintenance of anaesthesia should be with short-acting agents to expedite recovery.
- Emergencies and their devastating consequences can be prevented by education of carers, appropriate and serviced equipment and close monitoring

## Supplementary information

**Additional file 1.**

## Data Availability

is on request.

## Abbreviations

AAI-mode: Single chamber atrial pacing device
AASM: American Academy of Sleep Medicine
ACC: American College of Cardiology
AHA: American Heart Association
ANS: Autonomous nervous system
AS: Active Sleep
ATS: American Thoracic Society
BRUE: Brief resolved unexplained event
CCHS: Congenital central hypoventilation syndrome
DDD-mode: A dual chamber pacemaker
ECE1: Endothelin converting enzyme 1
ENT: Ear nose and throat
GDG: Guideline development group
HRS: Heart Rhythm Society
ICSD: International classification of sleep disorders
NPARM: Non-polyalanine repeat mutation
NPV: Negative pressure ventilation
NREM: Non rapid-eye movements sleep (Quiet Sleep)
PARM: Polyalanine repeat mutation
PHOX2B: Paired-like homeobox 2B
PPV: Positive pressure ventilation
QS: Quiet Sleep
REM: Rapid eye movements sleep (Active Sleep)
RET: Protein-altering mutations in receptor tyrosine kinase
ROHHAD: Rapid-onset obesity, hypoventilation, hypothalamic disorder and autonomic dysfunction
