# Supplementary material for: Guidelines for diagnosis and management of congenital central hypoventilation syndrome
Source: Orphanet J Rare Dis. 2020 Sep 21;15:252. doi: 10.1186/s13023-020-01460-2 (PMC7503443; doi:10.1186/s13023-020-01460-2)
Supplement: Supplementary file 1 — Additional file 1. [file 13023_2020_1460_MOESM1_ESM.doc]

**Appendix A**

The search strategies for congenital central hypoventilation syndrome (CCHS) are reproduced below. No language or date limits were applied. Given the relatively small number of records retrieved, no attempt was made to limit to under 18 years of age. Searching for specific age ranges is notoriously difficult and likely to lead to pertinent material being missed.

**MEDLINE (OvidSP –** [www.ovid.com](http://www.ovid.com/)**/)**

1950 to April week 2 2019

Searched on 26/04/2019

Retrieved 596 hits

And:

**MEDLINE In-process (OvidSP –** [www.ovid.com](http://www.ovid.com/)**/)**

April 23 2010

Searched on 26/04/2010

Retrieved 29 hits

Search Strategy:

1. congenital central hypoventilation syndrome.ti,ab.

2. central congenital hypoventilation syndrome.ti,ab.

3. ((ondine* or undine*) adj1 (curse or syndrome)).ti,ab.

4. phox2b.ti,ab.

5. Sleep Apnea, Central/cn [Congenital]

6. cchs.ti,ab.

7. congenital central alveolar hypoventilation.ti,ab.

8. central congenital alveolar hypoventilation.ti,ab.

9. or/1-8

10. animals/ not (animals/ and humans/)

11. 9 not 10 (601)

**EMBASE (OvidSP –** [www.ovid.com](http://www.ovid.com/)**/)**

1980 to week 16, 2019

Searched on 26/04/2019

Retrieved 655 hits

Search strategy:

1. congenital central hypoventilation syndrome.ti,ab.

2. central congenital hypoventilation syndrome.ti,ab.

3. ((ondine* or undine*) adj1 (curse or syndrome)).ti,ab.

4. phox2b.ti,ab.

5. ondine syndrome/

6. cchs.ti,ab.

7. central congenital alveolar hypoventilation.ti,ab.

8. congenital central alveolar hypoventilation.ti,ab.

9. or/1-8

10. animal/ not (animal/ and human/)

11. 9 not 10

**The Cochrane Library –** [www.thecochranelibrary.com](http://www.thecochranelibrary.com/)**/)**

April 2019

Searched on the 28/04/2019

Retrieved 23 hits (9 from CDSR, 14 from CENTRAL, 0 from DARE, HTA, NHS EED)

Search strategy:

#1 MeSH descriptor Sleep Apnea, Central, this term only with qualifier: CN

#2 "congenital central hypoventilation syndrome"

#3 "central congenital hypoventilation syndrome"

#4 (ondine* near/2 curse) or (ondine* near/2 syndrome) or (undine* near/2 curse) or (undine* near/2 syndrome)

#5 (phox2b)

#6 (cchs)

#7 "congenital central alveolar hypoventilation"

#8 "central congenital alveolar hypoventilation"

#9 (#1 OR #2 OR #3 OR #4 OR #5 OR #6 OR #7 OR #8)

**CINAHL - Cumulative Index to Nursing & Allied Health Literature (EBSCO –** [**www.ebscohost.com**](http://www.ebscohost.com/)**/)**

16/04/2019 update

Searched on 26/04/2019

Retrieved 73 hits

Search Strategy:

S1 (MH "Sleep Apnea, Central/FG")

S2 TI "congenital central hypoventilation syndrome" or AB "congenital central hypoventilation syndrome"

S3 TI "central congenital hypoventilation syndrome" or AB "central congenital hypoventilation syndrome"

S4 TI ondine* N3 curse or AB ondine* N3 curse

S5 TI ondine* N3 syndrome or AB ondine* N3 syndrome

S6 TI undine* N3 curse or AB undine* N3 curse

S7 TI undine* N3 syndrome or AB undine* N3 syndrome

S8 TI phox2b or AB phox2b

S9 TI cchs or AB cchs

S10 TI "congenital central alveolar hypoventilation" or AB "congenital central alveolar hypoventilation"

S11 TI "central congenital alveolar hypoventilation" or AB "central congenital alveolar hypoventilation"

S12 S1 or S2 or S3 or S4 or S5 or S6 or S7 or S8 or S9 or S10 or S11

S13 (MH "Animals+")

S14 (MH "Human")

S15 S13 and S14

S16 s13 not s15

S17 s12 not s16

**Appendix B.**

**Recognition criteria for the centres of reference for CCHS**

The European CCHS Network was tasked with developing a network of centres with specific expertise in the diagnosis and treatment of patients with CCHS. Delphi method was chosen by the members to elaborate and rank recognition criteria for such a centre of reference for CCHS. Following two rounds of the Delphi survey, representatives of the EU-CHS Consortium and knowing the results of both rounds, decided to divide recognition criteria into 2 categories: A: obligatory and B: recommended. Recognition criteria for a centre of reference for CCHS proposed by the EU-CHS consortium are in line with EURORDIS position paper on “Centres of Expertise and European Reference Networks for Rare Diseases” (2008). Median values are given.

| The unit | First round | Second round | Ranking proposal |
| --- | --- | --- | --- |
| Regularly manages CCHS patients | 16 | 17 | A |
| Regularly manages home ventilation | 16 | 16 | A |
| Has regular access to intensive care in the hospital | 15 | 15 | A |
| Regularly performs cardio-respiratory recordings (PCO_2_) | 13 | 14 | A |
| Adopts multidisciplinary approach | 11 | 12 | A |
| Involves families in the patient care | 11 | 11 | B |
| Regularly performs polysomnography | 12 | 11 | B |
| Has access to genetic studies | 11 | 10 | B |
| Provides training and courses to families | 9 | 10 | B |
| Has flexible in-patient facilities and visiting | 8 | 9 | B |
| Provides training and courses to health professionals | 8 | 8 | B |
| Has a written discharge protocol | 8 | 7 | B |
| Has its own written guidelines for diagnosis and management | 8 | 7 | B |
| Is involved in clinical research projects | 8 | 4 | B |
| Conducts clinical research projects | 4 | 3 | B |
| Has published on CCHS in international revues | 4 | 3 | B |
| Has published on CCHS in national revues | 3 | 3 | B |
